# Corpse management of the invasive Argentine ant inhibits growth of pathogenic fungi

**DOI:** 10.1038/s41598-019-44144-z

**Published:** 2019-05-20

**Authors:** Jenni Kesäniemi, Janne J. Koskimäki, Jaana Jurvansuu

**Affiliations:** Ecology and Genetics Research Unit, University of Oulu, FI-90014 Oulu, Finland

**Keywords:** Behavioural ecology, Invasive species

## Abstract

A dead conspecific poses a potential pathogen risk for social animals. We have discovered that Argentine ants (*Linepithema humile*) prevent spread of pathogenic fungi from corpses by depositing the dead to combined toilet and refuse areas and applying pygidial gland secretion on them. The presence of a corpse in a nest increases this secretion behaviour. We identified three fungi growing on Argentine ant corpses. Growth of the Argentine ant pathogen *Aspergillus nomius* and the plant pathogen *Fusarium solani* on corpses was inhibited as long as the ants were constantly attending them as the ant anal secretion only delayed germination of their spores. In contrast, the effect of the ant anal secretion on the human pathogen *Aspergillus fumigatus* was much stronger: it prevented spore germination and, accordingly, the fungus no longer grew on the treated corpses. The Argentine ants are one of the world’s worst invasive alien species as they cause ecological and economical damage in their new habitats. Our discovery points at a novel method to limit Argentine ant colonies through their natural fungal pathogens.

## Introduction

Social immune system is a term used to describe co-operative behaviour of social animals to reduce vulnerability to disease transmission that arises from living in a genetically homogenous closely interacting group^1^. For example, management of corpses, faeces, and food waste is part of the social immune system to improve nest hygiene. Corpse-induced behavioural responses in insects include corpse removal from the nest, burial, cannibalism, avoidance, and combinations of these^2,3^. Undertaking responses depend also on disease and developmental state of the cadaver as well as nesting and feeding habits of the insect^4^. Corpse management aims to prevent growth and spread of pathogens and parasites: for example, it has been shown in a laboratory experiment that a single fungal infected cadaver can be fatal to the whole ant colony^5^. In some highly socially organised insects corpse management has been suggested to be separate from the other basic cleaning activities, such as disposing faeces and food remains. For example, some ant species remove corpses from nest more quickly and farther away than other objects^6,7^, foreign corpses are treated differently to nestmates’ corpses^8^, and faeces and corpses are deposited into separate areas^9^.

The Argentine ant is listed in the International Union for Conservation of Nature’s 100 world’s worst invasive alien species, because it has spread from South America to all over the world during the past 160 years and is able to harm ecosystems by displacing native species^10–12^. The success of the invasive Argentine ants has been attributed mostly to their lack of territoriality as the ants mix freely between nests and can hence form extensive supercolonies spanning thousands of kilometers^13,14^. Whereas introduced Argentine ants from two different continents may show no hostility^15^, the native South American Argentine ants that form much smaller^16^ and short-lived^17^ colonies display aggression among colonies^18^. The unicolonial social structure enables high population densities^19^, which together with low genetic diversity^20^ and relative few immune genes^21^ may predispose the invasive Argentine ants to pathogens. Thus, hygiene behaviour is expected to be especially important for their survival.

Argentine ants deposit food waste and dead bodies onto refuse piles^22^ yet their defecating behaviour has not been studied. We discovered that Argentine ants defecate and secrete pygidial gland content onto the corpses at the refuse piles and that this behaviour inhibits growth of pathogenic fungi.

## Results

### The Argentine ants deposit corpses and faeces on the same area

**Figure 1:**
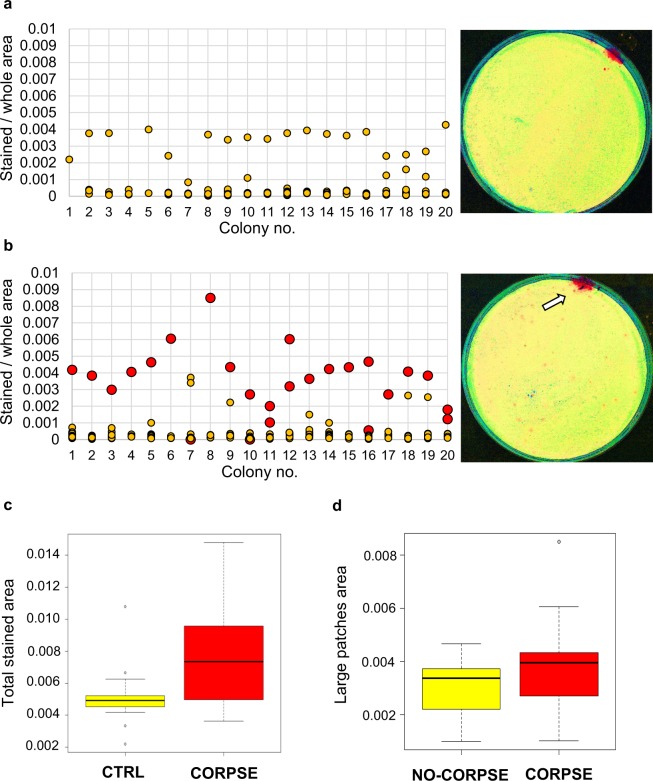
The Argentine ants have combined toilet and refuse pile areas. (**a**) Argentine ants defecate on dedicated toilet areas. Worker ants were plated on plaster-bottom petri dishes (N = 20) and they were given blue-coloured sugar solution for food. Plates were photographed four days after and the area of each blue patch was measured and blotted for each plate (colony). The false-colour image of a presentative plate shows toilet area in red. (**b**) The Argentine ants deposit corpses on the toilet areas. Ants were plated on plaster-bottom petri dishes (N = 20) and were given blue-coloured sugar solution for food and the next day one ant was killed and the corpse was immediately placed back to the middle of the nest plate. Three days later the plates were photographed and the stained areas were measured and the location of the corpse was recorded and blotted for each plate (colony). In a blot the yellow circles denote toilet patches and red circles are toilet patches with a corpse in them. In colonies #10, 11, 12, 16, and 20 the corpse was chopped into two pieces and deposited into two different toilet areas. In colony #7 the corpse was outside of toilet areas and it is marked with red circle at zero area. In the presentative false-colour image, the toilet areas are in red and a white arrow indicates the location of the corpse. (**c**) The presence of a corpse in a nest induces anal secretion. Total area of all the blue patches were calculated for each nest without (yellow, CTRL) and with a corpse (red, CORPSE). (**d**) Large blue patches (>0.001 stained/whole area) with a corpse (red, N = 22) were significantly larger than patches without a corpse (yellow, N = 33). In the box plots, the black line represents median value, values inside the box cover first and third quartile (interquartile) range, the whisker show values up to 1.5 times the interquartile range, and grey circles are individual values beyond the whisker range (outliers).

We plated 20 ants to plaster-bottom petri dishes and fed them with blue-coloured sugar mix. After four days we measured the sizes of the blue stained areas they left on the plaster. We repeated the experiment 20 times. The ants had clearly a preferred toilet area at the periphery of the plate as indicated by the large blue stained areas (1–2 toilets per plate, size >0.001 stained/whole area, that is, the proportion of stained area from the whole surface are of the plate), although several tiny patches were also visible (total number of blue patches per plate: mean = 9, SD = 8.6) (Fig. 1a).

As it was earlier shown that black garden ants (*Lasius niger*) have separate toilet area and waste pile to where the corpses were laid^9^, we studied next where Argentine ants put their dead. We modified the previous experiment by killing one of the ants a day after plating them, placing it immediately back to middle of the plate, and recording its location three days later. The experiment was repeated 20 times. We found that the corpses were in the large (>0.001 stained/whole area, 1–2 per plate) toilet areas and not at random locations (Fisher exact test p-value < 0,00001) (Fig. 1b). We saw the same effect with ants fed with fluorescence dye sugar mix instead of blue dye (see Supplementary Fig. 1).

When the ants were given a corpse they produced larger total area of blue patches than when there was no corpse in a plate (N = 40, Mann-Whitney U = 99, p = 0.006, Fig. 1c). However, when only the larger blue areas were considered (>0.001 stained/whole area, N = 55), there was no difference in their sizes between the colonies that were given a corpse (Fig. 1a) or not (Fig. 1b) (Mann-Whitney U = 326, p = 0.408). Yet the large blue patches with a corpse (N = 22) were significantly larger than patches without a corpse (N = 33) (Mann-Whitney U = 515, P = 0.009, Fig. 1d). These results suggest that in the presence of a corpse, ants increase defecation and/or secretion of anal gland products, specifically to the area where the corpse is (see Supplementary video 5 and Supplementary videos 1–4 for prior corpse processing behaviour).

### Placing corpses to toilets inhibits fungal growth on them

**Figure 2:**
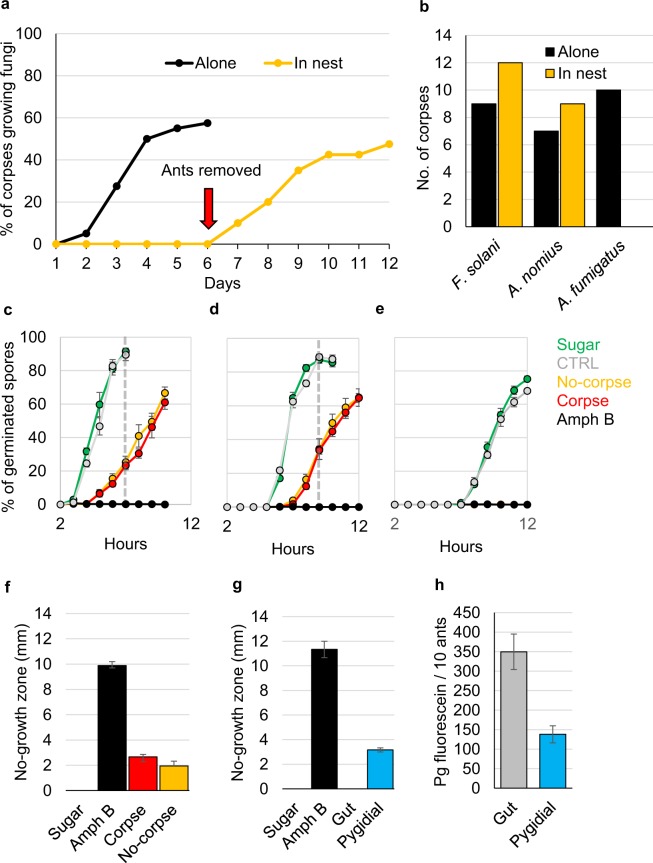
The Argentine ant anal secretion inhibits mould growth. (**a**) Worker ants were plated on plaster-bottom petri dishes (N = 40 plates) and were given sugar solution for food. The next day two of the ants were killed and the first corpse was put on an empty plaster-bottom petri dish and the second back to the nest plate. After six days, ants were removed from the nest plate (red arrow) and the appearance of visible fungal growth was recorded for six more days. The blot shows the percentage of corpses visibly growing fungi for lone corpses (black, Alone) and corpses in the nests (yellow, Nest). (**b**) *F*. *solani*, *A*. *nomius*, and *A*. *fumigatus* were identified from the corpses. The bars show how many corpses were visibly growing each fungus in lone (black, at six days) or nest (yellow, at 12 days) conditions. Effect of the isolated Argentine ant gaster-liquid on spore germination was measured for (**c**) *F*. *solani*, (**d**) *A*. *nomius*, and (**e**) *A*. *fumigatus*. Gaster-liguid was collected from ants that had two corpses (red) or none (yellow) in a nest. Spores were plated on nutrient agar plates and 1 µl of gaster-liquid, 10% sugar solution (green), or fungicide Amphotericin B (black) was added to three spots on a plate and percentages of germinated spores were recorded every hour for 12 hours. Grey dashed lines indicate the time points for statistical analysis. The experiment was repeated three times (N = 9, variation is ± SE). (**f**) Gaster-liquid prevented germination of *A*. *fumigatus* spores and after 24 hours no-growth zones (mm of diameter) were measured for the gaster-liguid (corpse: red bar, no-corpse: yellow bar), 10 % sugar, and Amphotericin B (black bar) treated spots (N = 9, variation is ± SE). (**g**) Pygidial gland but not gut-derived secretion inhibited *A*. *fumigatus* growth. Gut and pygidial gland content were collected separately and used in *A*. *fumigatus* spore germination test. No-growth zone was measured 24 hours after the application of the samples. The experiment was repeated three times using two different isolations (N = 6, variation is ± SE). (**h**) Gut secretion liquid had twice as much of food-derived fluorescein than pygidial gland secretion. Three separate isolations from 10 ants were measured (N = 3, variation is ± SE).

We noticed that when the ants are starving (were given only water) or fed with neonicotinoid neurotoxin (Imidacloprid) they did not pile or process the corpses and these unmanaged corpses started to grow fungi in a few days (see Supplementary Fig. 2). These observations indicated that fungal growth is inhibited from the corpses placed at the toilet areas. To test this idea we plated 22 ants on plaster-bottom petri dishes and a day after we killed two of them: the first corpse was placed to an empty new plate and the second corpse was put back to the nest plate. The corpse in the nest plate was kept with the living ants for six days after which the ants were removed. We recorded how many days after the ants were killed would fungus visibly grow on the corpses. The experiment was repeated 40 times. When a corpse was alone it started to grow fungus, on average, three days after death, whereas corpses in the nests did not grow fungi as long as there were living ants present (Fig. 2a). When the ants were removed, the corpses in the nests started to grow fungus in one day even if the corpses were in the toilet areas. Inhibition of fungal growth on corpses in the nests and alone was significantly different (inhibition shown with survival analysis with number of days until fungal growth from the corpses: Cox’s proportional hazard model, hazard ratio HR = 0.40, p = 0.002, see Supplementary Fig. 3). The overall percentage of fungi growing corpses remained somewhat lower in the nests (47.5%) than in the empty plates (57.5%), yet the difference is not statistically significant (Chi^2^ with Yates correction = 0.4511, p-value = 0.5018).

### Fungal identification

**Figure 3:**
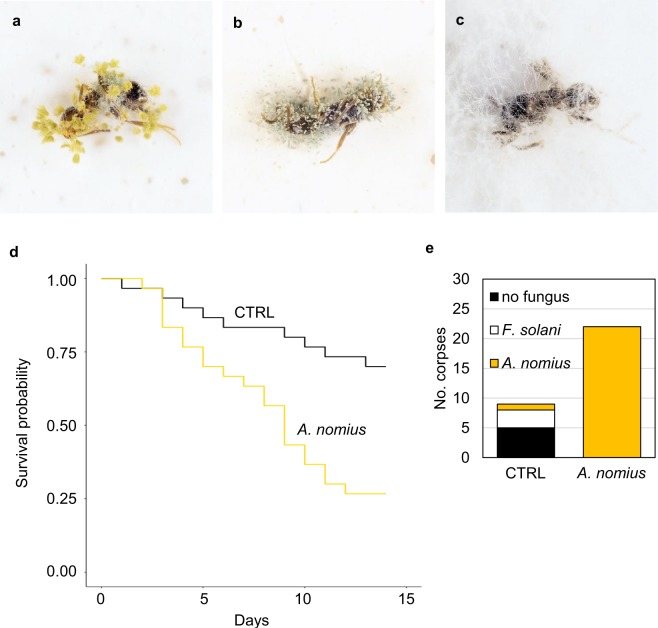
*A*. *nomius* is an Argentine ant pathogen. Presentative photographs of Argentine ant corpses growing (**a**) *A*. *nomius*, (**b**) *A*. *fumigatus*, and (**c**) *F*. *solani*. Effect of *A*. *nomius* spores on ant survival was tested by applying spores onto the ant thorax. (**d**) Survival of infected (*A*. *nomius*, N = 30) and control (CTRL, N = 30) ants were recorded for 14 days. Difference in survival between the treatments was calculated using the Cox’s proportional hazard model. The corpses were surface sterilized the day they died and visible appearance of fungal growth on corpses was recorded for at least 7 days. (**e**) All *A*. *nomius* infected (N = 22) corpses grew *A*. *nomius* whereas over half of the control corpses (N = 9) grew no fungus at all.

The corpses were growing three visibly different fungi, which were identified by internal transcribed spacer (ITS) sequencing to be *Aspergillus nomius* (yellow conidia, Fig. 3a), *Aspergillus fumigatus* (blue conidia, Fig. 3b), and *Fusarium solani* (completely white appearance, Fig. 3c). Interestingly, frequencies of the three fungi growing on corpses were dependent on whether the corpses were alone or in the nest (Fig. 2b, Freeman-Halton Fisher exact test p-value = 0.003152). The corpses treated with anal secretion by their nestmates never grew *A*. *fumigatus* but still readily grew *A*. *nomius* and *F*. *solani*, whereas all three fungi grew in corpses that were alone.

### Argentine ant pygidial gland secretion inhibits fungal spore germination

To tests whether Argentine ant anal secretion inhibits fungal spore germination, we collected gut and gland contents by gently squeezing the ant’s gaster. Because in the previous experiment we saw that the presence of a corpse increased the secretion behaviour, we isolated gaster-liquids from ants that either had no corpse or had two corpses in the nest. We used fungicide Amphotericin B as a negative and 10% sucrose solution and no-treatment as positive controls. We plated *F*. *solani*, *A*. *nomius*, and *A*. *fumigatus* spores onto nutrient agar plates and added 1 µl of the gaster-liquid samples and controls to the plates. We were not able to use the common disc diffusion assay as the amount of liquid extracted from the ants was not sufficient. Percentage of germinated spores was recorded for each fungus every hour until the germination percentage at the control samples reached plateau (Fig. 2c–e). We repeated the experiment three times and had triplicates of each treatment (gaster-liquids and controls) on each plate. Additionally, the area of inhibition was measured for *A*. *fumigatus* samples 24 hours post-plating (Fig. 2f). The gaster-liquid delayed the start of the germination of *F*. *solani* and *A*. *nomius* spores by about two hours. As the germination rate of spores treated with gaster-liquid collected from ants housed with or without corpses was similar, these samples were combined as the “gaster-liquid” treatment. This also suggests that the production of the effective substance in the gaster-liguid is not induced by the presence of a corpse. Additionally, the two positive controls (sugar and no-treatment) were combined for the analysis (Fig. 2c,d, see Supplementary Table 1). Spore germination rates of these two groups were compared at the time point when the positive control samples had reached a plateau. For *F*. *solani*, spore germination percentage was significantly lower for the spores treated with gaster-liquid (7 hours post-plating, p < 0.001, 24% and 91% for gaster-liquid and control, respectively). Similarly, for *A*. *nomius*, spore germination percentage was lower  for the gaster-liquid treated spores (8 hours post-plating, p < 0.001, 34% and 88% for gaster-liquid and control, respectively) (Table 1). The germination of *A*. *fumigatus* was completely inhibited by the gaster-liquid since we detected no germinated spores during the 8 hour observation period when about 70% of spores had germinated in the controls (Fig. 2e). After 24 hours we saw a no-growth zone where the gaster-liguid was applied indicating that the effect was permanent (Fig. 2f). On average, 250 ng of Amphotericin B produced 10 mm and gaster-liquid 2.7 mm diameter no-growth zone.

**Table 1:** GLM results showing the effect of gaster-liquid treatment (in comparison to control samples) on the spore germination of *F*. *solani* and *A*. *nomius*.

*F*. *solani*	Estimate	SE	t	p
Intercept	103.79	3.71	27.96	<0.001
Gaster-liquid	−67.00	2.46	−27.22	<0.001
Plate	−6.25	1.53	−4.08	<0.001
***A***. ***nomius***	**Estimate**	**SE**	**t**	**p**
Intercept	95.69	4.98	19.21	<0.001
Gaster-liquid	−54.28	3.41	−15.89	<0.001
Plate	−3.71	2.11	−1.76	0.088

Since the collected gaster-liquid consist mainly of the content of rectum and the pygidial gland, we decided to collect the two secretions separately and test them against *A*. *fumigatus* spores using a growth inhibition test (Fig. 2g). We used the same controls as above: sugar and Amphotericin B. Gut liquids did not inhibit fungal spore germination at all (no-growth zone of 0 mm) whereas pygidial gland content produced similar effect with original gaster-liquid (no-growth zone of 3.1 mm).

Since pygidial gland secretion, not gut liquids (i.e., faeces), inhibited the fungal germination, we examined the distribution of food-derived fluorescence dye into gut and pygidial gland in order to see whether increase in application of pygidial gland secretion could contribute to plaster staining in the behavioral experiment (Fig. 1c,d). Although significantly more fluorescein was in the gut secretion than in pygidial gland (student’s t-test p-value = 0.0137), the amount was only 2.5 times higher and thus also the pygidial gland secretion would be visible in dye feeding experiments and would contribute  to the size of the stained areas.

### *A*. *nomius* kills Argentine ants

We tested by trial experiments the sensitivity of the ants to the three fungi we identified (see Supplementary Fig. 4). Because the preliminary results suggested that *A*. *nomius* was the most effective in killing the ants, we tested its effect on the ant survival by topical application of *A*. *nomius* spores (Fig. 3d). We used 0.1% tween as a control treatment. We followed survival of the ants in individual plates for 14 days. *A*. *nomius* treatment increased mortality significantly in comparison to control treatment (lowered survival probability shown with Cox’s proportional hazard model, N = 60, HR = 3.541, 95% Cl for HR 1.618–7.747, z = 3.165, and p = 0.00155). We surface sterilised the corpses on the day of their death and over half of the control corpses did not grow fungus during the seven day observation period (Fig. 3e). However, all the *A*. *nomius* infected corpses showed *A*. *nomius* growth, on average, in 1.36 days suggesting that their death was due to *A*. *nomius* infection.

## Discussion

Argentine ants start carrying dead nestmates to refuse piles when cuticular “life-associated” chemicals, dolichodial and iridomyrmecin, have disappeared^22^. These volatiles are secreted from pygidial gland and Argentine ants use them also as defensive compounds^23^ and trail pheromones^24^. Argentine ants have four distinct anal endocrine glands: Pavan’s, Dufour’s, venom, and pygidial gland^25^, from which the pygidial gland is the largest^25^. Iridomyrmecin is major product of pygidial gland^23^ and it has been shown to be weakly antibacterial^26^. Beetle pygidial gland secretion has been described to inhibit fungal growth^27–29^. However, fungicidal properties of ant pygidial gland chemicals have not been reported before.

Ants generally inhibit fungal and bacterial growth on their surface by self- and allo-grooming^30,31^, metapleural^32^ and poison gland secretion^33^, and symbiotic bacteria^34,35^. Interestingly, Argentine ants, unlike many other ant species, were found to lack antibacterial substances on their surfaces^36^. Moreover, *Linephitema melleum*, a congener of Argentine ant, has been shown to spread antibiotic metapleural secretion only on themselves and not on nestmates, brood, or queens as some other ant species do^37^. Thus, if Argentine ants have to rely on grooming to keep bacteria and fungal spores away, then contamination risk from corpses is high. Furthermore, since Argentine ants are predators^38,39^ they bring other insect species into the nest, which is an additional source of pathogenic fungi. Hence, as the Argentine ant pygidial gland secretion has anti-fungal properties, combining the refuse pile and toilet area provides simultaneous sanitation of food waste, corpses, and faeces. Other corpse and waste management associated processes, such as, chopping up the corpses, eating them, and collecting other nest materials on the refuse piles will also inhibit fungal growth^3,40,41^.

In nature ants have refuse piles inside or/and outside the nest and some species have dedicated workers to manage the waste^42–44^. *Eurhopalothrix heliscata* (Myrmicinae) builds a waste chamber inside the nest, yet in an artificial nest they deposit waste on a corner of a foraging area^45^ like Argentine ants^22^. The red ants (*Myrmica rubra*) dispose corpses outside the nest but when they are placed into confined artificial nest they scatter corpses around the area^2^. Thus, although our experimental set-up was artificial, the results will likely reflect ants’ natural behaviour.

We identified three fungi growing from the dead Argentine ants: *F*. *solani* belongs to a species complex best known as plant pathogens^46^, *A*. *nomius* is an aflatoxin-producing entomopathogen^47–50^, and *A*. *fumigatus* is one of the most common pathogenic fungus in immune compromised humans^51^. All these opportunistic fungi are widespread in nature and especially *Aspergillus* spp. are commonly associated with social insects and their nests^52–55^. According to our results, *A*. *fumigatus* was the most sensitive to the effects of Argentine ant pygidial gland secretion as the gland secretion prevented the spore germination and the fungus never grew on the bodies at the refuse piles. The inhibitory effect of the pygidial gland secretion on Argentine ant pathogen *A*. *nomius* and plant pathogen *F*. *solani* was transient both *in vivo* and *in vitro* assays. Hence, inhibiting the growth of *A*. *nomius* and *F*. *solani* requires constant application of pygidial gland secretion on the corpses. Consistently, the presence of a corpse increased stained areas especially at the toilet/refuse piles where the corpse was, suggesting that corpse may have induced pygidial gland secretion behaviour. We cannot separate in our experiments faeces and pygidial gland-derived staining, since the food-derived dye was able to cross to pygidial gland content as well. However, as pygidial gland secretion has been shown to attract nestmates as a trail pheromone^24^ and defence substance^23^, its application on corpses could create virtuous cycle, whereby more ants gather to the site, which leads to increased pygidial gland secretion and thus more efficient fungal growth inhibition.

Argentine ants are notoriously difficult to eradicate due to their non-territoriality, large colony sizes, and existence of several queens per nest^56^. Our results suggest that Argentine ants secrete pygidial gland chemicals on toilet/refuse piles to inhibit growth of pathogenic fungi on corpses and other waste. If this behaviour is crucial for nest hygiene, it might open a mechanism to lower the population fitness of this destructive invasive pest by neutralising the anti-fungal component(s) of the pygidial gland secretion.

## Methods

### Ants

The Argentine ants were collected in April 2011 from the European Main supercolony in Catalonia, Spain^57^. The ants were kept in artificial nest in Sanyo climate chambers set to 14 hours of light in 27 °C and 10 hours of dark in 20 °C, and air moisture between 40–60%. Ants had water all the time and were fed two times per week with honey and cockroaches. For experiments ants were removed from the artificial nest to wetted plaster-bottom 9 cm diameter petri dishes and they were given (50 µl) 10% sucrose solution or 2.5% blue food colouring (Brilliant Blue FCF, Dr. Oetker) in 10% sucrose solution.

### Behavioural experiments

For toilet localisation experiment, ants were removed after four days of plating and plates were photographed. For corpse management experiment, one of the ants was killed by squeezing with forceps one day after plating and placed immediately back to the middle of the nest plate. Three days after the plate was photographed. The Fisher exact test compared the observed (19 bodies in large toilets, 0 in small patches, and 1 outside toilets) and expected values based on probabilities of corpse being randomly either in or outside of a toilet area. The experiment was repeated 20 times.

For fungus growing experiment 22 ants were plated and a day after plating two ants were tweezed to death, one was placed directly to a new wetted plaster-bottom petri dish, and the other corpse was placed back to middle of the nest plate. After six days, ants were removed from the nest plate. Fungal growth on bodies was inspected for 12 days (time until fungal growth was used in the survival analysis testing for the inhibitory effect of ant gaster-liquids, see Fig. 3 in Supplemental material). The experiment was repeated 40 times.

### Fungi

Fungi were isolated from ant corpses and subcultured on potato dextrose agarose (PDA) plates. Axenic cultures of each species were used for the experiments and species-level identification. Fungal cultures were maintained at the same conditions as the ants. Spores were collected by submerging fungus growing on a PDA plate into 10 ml of sterile distilled water containing 0.1% Tween-20 (v/v) to release the spores. The spore suspensions were filtered through one layer of Miracloth (EMD Millipore, cat. no. 475855, pore size of 22–25 µm). For the experiments, freshly-collected spore suspensions were counted and adjusted for specific spore concentration (1 × 10^6^–2 × 10^7^ spores/ml). Suspensions were stored at 4 °C and used in the experiments during the same day.

### Identification of ant associated fungi

Fungal samples were collected into 15 µl of sterile water, boiled for 5 min, and then used as a template for PCR. To identify the fungi, region of the fungal rRNA gene repeat, Internal Transcribed Spacer (ITS2), was amplified using fungal specific primers fITS7^58^ and ITS4^59^. PCR was performed in 20 µl final volume, containing 1X Phusion HF Buffer, 200 µM of dNTPs, 0.5 µM of both primers, and 0.02U/µl of Phusion high-fidelity DNA polymerase (ThermoFisher). The following PCR protocol was used: 98 °C for 3 min, followed by 35 cycles of 98 °C for 10 s, 57 °C for 15 s, and 72 °C for 30 s, with a final elongation of 72 °C for 7 min. For each of the three fungal species, two representative samples were sequenced: one collected directly from a dead ant’s surface, and another from an axenic subculture from PDA plates. PCR products were sequenced with fITS7 and ITS4 primers at Eurofins. The fungi were identified by Blastn search of the National Center for Biotechnology Information database.

### Spore germination assay

Spores were counted and diluted into 1 × 10^7^/ml for *F*. *solani*, 2 × 10^7^/ml for *A*. *nomius*, and 3.5 × 10^7^/ml for *A*. *fumigatus*. Spore solutions (100 µl) were plated on nutrient broth agar plates (Scienova) and incubated at 30 °C for either two or four hours before test solutions were added (due to different germination speeds of the fungi). On each fungal spore plate, 1 µl of the gaster-dilutions, Amphotericin B (250 µg/ml), or 10% sucrose solution was applied to three separate spots (i.e., three replicates). Spore germination, i.e., existence of visible germ tube, was recorded every hour for the next 8 hours, each time examining 100 spores under light microscope. 24 hours from the plating the diameter of no-growth zone was measured for *A*. *fumigatus* plates. Experiments were repeated three times.

The effect of the treatment (gaster-liquid or positive control) on the spore germination percentage was tested using Generalized Linear Model (GLM, with Gaussian distribution) using R, controlling for the variation among plates with using the plate information as a factor in the analysis. The gaster-liquid treatment comprised of samples from ants of nest plates with or without a corpse present, and positive controls were combined samples of sucrose treatment and control without any treatment. Spore germination was compared at the time point when the positive control samples had reached a plateau, i.e. 7 and 9 hours after the plating the spores for *F*. *solani* and *A*. *nomius*, respectively.

### Anal-liquid collection

A day before the collection 50 ants were plated per plaster plate with 10% sucrose solution for feed. In half of the nests two ants were killed and placed to the nest two to six hours before the liquid collection. Liquid was collected from ants by squeezing their gaster into drop (2 µl) of sterile water. Gaster-liquid from about 200 ants (in total about 2 µl) was collected into a 20 µl of water and frozen at −20 °C until use. We standardised the amount of gaster-liquid collected at separate times by measuring absorbance at wavelength of 275 nm.

To collect gut and pygidial gland contents separately, ant gaster was squeezed and when gut (ventral opening) and/or pygidial gland (dorsal opening) secretion droplets were visibly separable under microscope they were collected to separate drops (1 µl) of sterile water by touching the surface of the water drop with the secretion droplet. Argentine ant gaster has been shown to contain several gland openings ventrally (i.e., Pavan’s, Dufour’s, and venom)^25^ and thus we cannot rule out contents from these glands in the gut fraction.

### Fluorescein distribution measurements

Ants were plated on plaster-bottom petri dishes and given 10 µg/ml fluorescein (Fluka) in 10% sucrose solution to feed. After two days gut and pygidial gland secretions were squeezed from 10 ants and diluted into 50 µl of water. Isolation was repeated three times. Fluorescein concentration in the samples were measured with plate reader (485 nm excitation/520 nm emission, Victor PerkinElmer) and the amount of fluorescein was calculated from linear standard curve.

### Ant survival experiments

Effect of exposure to *A*. *nomius* spores on ant survival by surface application was studied by pipetting a 1 µl drop of either 1.6 × 10^7^/ml *A*. *nomius* spores or 0.1% Tween-20 onto the ant thorax. A day before the experiment, 60 ants were plated onto their own plaster-bottom petri dishes with 50 µl of 10% sucrose solution. 30 ants were treated with the spore solution and 30 ants with the control solution. Survival of the ants were recorded for 14 days and each day the dead were removed from the nest plate, surface sterilized^60^, and fungal growth from the corpses was recorded until the end of the experiment or at least 7 days.

Differences in the survival of ants (days until death) treated with *A*. *nomius* spore solution and control solution was analyzed using survival analysis (the Cox’s proportional hazard model: surviving individuals were included in the analysis, but scored as separate from the dead ants), with the R package Survival^61^ (treatment was coded as 1 = ctrl and 2 = *A*. *nomius* spores).

### Pictures and videos

Pictures were taken with Nikon D3300 camera with AF-P Nikkor 18–55 mm objective or with Olympus OMD EM1 camera with M. Zuiko Digital ED 60 mm f2.8 macro objective. Picture and video analysis and processing were done with Olympus viewer software, iMovie, ImageJ (Fiji), and Corel PaintShopt Pro X9.

### Statistical analysis

Statistical analysis was done with R v.3.5.0 and IBM SPSS Statistics 24.

## Supplementary information

Supplementary data

Supplementary video 1

Supplementary video 2

Supplementary video 3

Supplementary video 4

Supplementary video 5

## Data Availability

The datasets generated during the current study are available from the corresponding author on reasonable request.
