# Supplementary material for: Corpse management of the invasive Argentine ant inhibits growth of pathogenic fungi
Source: Sci Rep. 2019 May 20;9:7593. doi: 10.1038/s41598-019-44144-z (PMC6527551; doi:10.1038/s41598-019-44144-z)
Supplement: Supplementary file 1 — Supplementary data [file 41598_2019_44144_MOESM1_ESM.pdf]

Corpse management of the invasive Argentine ant inhibits growth of pathogenic fungi

Kesäniemi Jenni, Koskimäki Janne J. & Jurvansuu Jaana

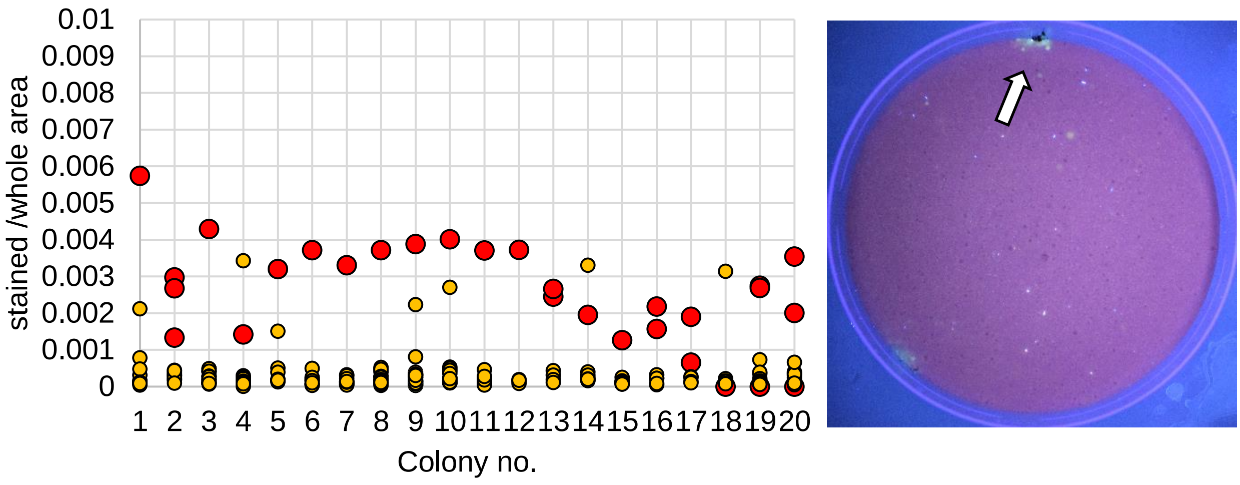

Figure 1. The Argentine ants deposit corpses on toilet areas. Ants were plated on 20 petri dishes (20 ants/plate)and fed with 100 ug/ml of riboflavin (ThermoFisher) in 10 % sugar solution. One day after plating one ant was killed and placed back to the middle of the nest plate. Pictures were taken three days later under UV (352 nm), the areas of fluorescence patches was measured, and the location of the corpse was recorded and blotted. Toilet areas without a corpse are in yellow circle and with corpse in red circle. The corpse may be in many pieces and thus in many toilet areas. If the body was outside a toilet area, it is indicated by red circle at zero area. In the photograph of the plate, toilet areas fluorescence in yellow and the location of the corpse is indicated by a white arrow. The ants had clearly a preferred toilet area at the periphery of the plate as indicated by the large yellow areas (1-3 toilets per plate, size > 0.001 units), although several tiny patches were also visible (total number of yellow patches per plate: mean = 15, SD = 10). According to the Fisher exact test, the corpses were not at random locations (p-value < 0,00001) but in the large toilet areas.

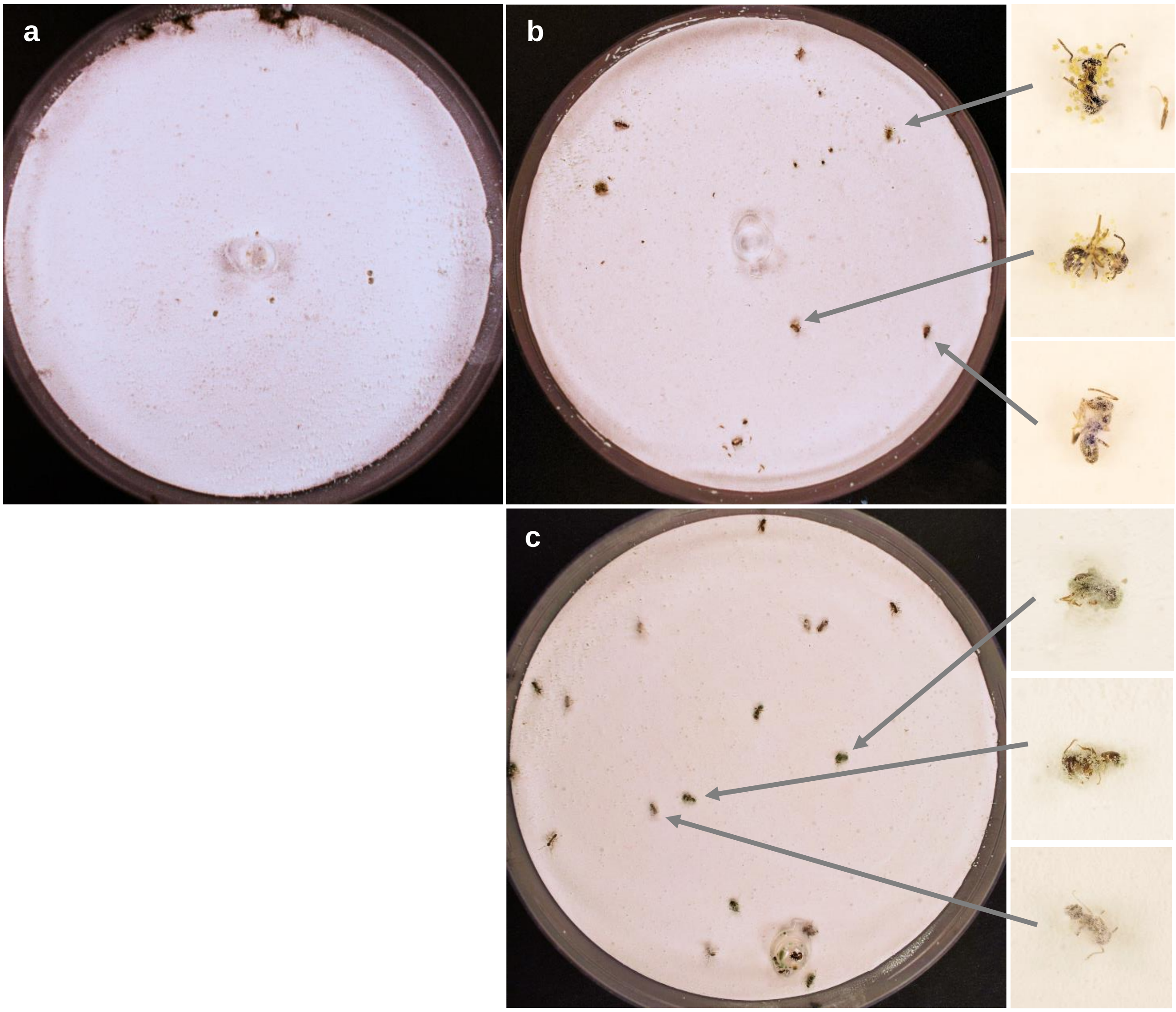

Figure 2. Unwell Argentine ants do not process the corpses into refuse piles. a) Normally Argentine ants collect corpses into refuse piles (the three black masses on the top of the plate without fungal growth). b) Starved ants fed only with water and c) ants fed with 0.0005 % imidacloprid (Bayer) in 10 % sucrose do not pile their corpses. Unprocessed corpses start to grow mould in 3-4 days after death (insets). The corpses in the picture are 1-7 days old. Starved ants die, on average, in 10 days and imidaxloprid-poisoned ants in 17 days.

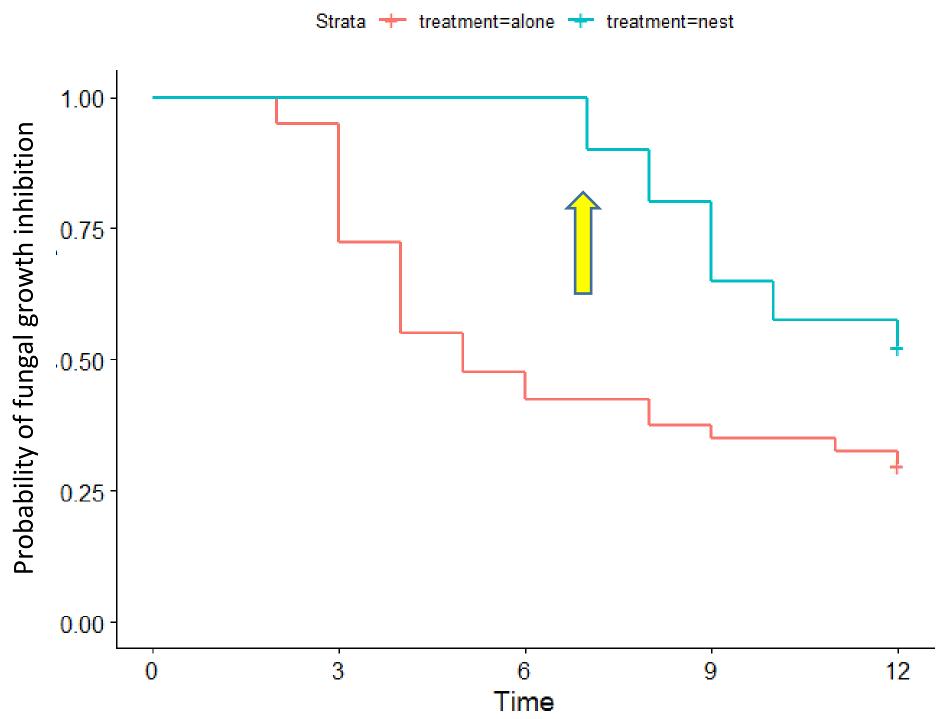

Figure 3. Inhibition of fungal growth on corpses in the nests and alone was significantly different. Survival analysis was used to show the inhibition of growth by testing whether the time (in days) from death until fungal growth differed in the two treatments. Cox's proportional hazard model was calculated using the data from Fig. 2a (including six additional data points for alone-treatment). Corpses alone are in red, corpses in nests are in blue, and yellow arrow indicates the time point when living ants were removed from the nest experiments. When ants were present in the nest, the fungal growth was delayed (Hazard ratio HR=0.40 and p-value=0.002).

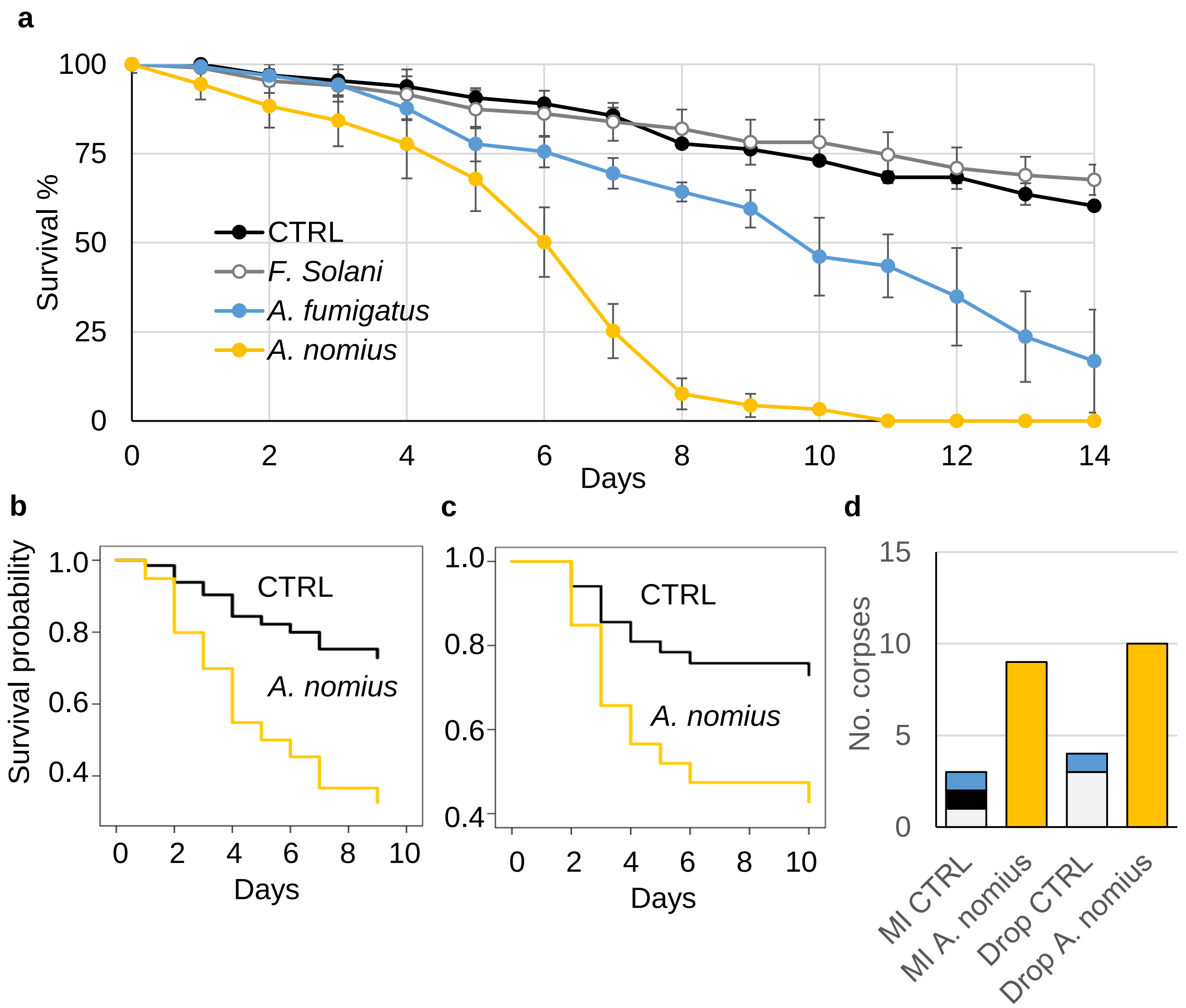

Figure 4. Sensitivity of the Argentine ants to the identified fungi. a) Subcultures of *F. solani* (grey), *A. fumigatus* (blue), or *A. nomius* (yellow) were transferred to new PDA plates and grown for seven days until they covered approximately 75 % of the plate. 20-40 ants were plated on the fungal plates or on an empty PDA plate (CTRL, black) with 10 % sucrose solution (50 µl). Survival of the ants was recorded every day for 14 days. The experiment was repeated for two times for control plate, three times for *F. solani,* and four times for both *Aspergillus* plates (experimental variation is shown by ±SE). Ants were infected with *A. nomius* spores either by b) microinjecting (MI) 200 spores or same volume of 0.1% tween (CTRL) into the gaster, or c) pipetting a 1 µl drop (Drop) of either spore

mixture (1.6 x 10^4 spores) or 0.1% tween (CTRL) onto the thorax. Injections were performed as described by Viljakainen *et al*.[1]. Survival of infected (*A. nomius*) and control (CTRL) ants were recorded for 10 days. Each experiment had at least five ants and was repeated three times (N=15 per treatment). Ants that died one day post-injection were omitted from the analysis. Although the experiments indicated that exposure to *A. nomius* reduced survival of the ants, the results were not statistically significant due to small sample size, pooling of individuals on the same plate, and only three replicates (drop data: N=30, HR=3.739, z=1.72, P=0.086, plate variance 0.338, plate SD=0.582; microinjection: N=27, HR=3.151, z=1.07, P=0.28, plate variance=0.919, plate SD=0.958). The statistics were calculated using survival analysis with the Coxme R package2 and with treatment (A. nomius or CTRL) as a fixed factor and replicate (i.e. plate) as a random factor. Dead ants were removed from the nest plates to new plate and after 11 days the type of fungi visibly growing on the corpses were identified morphologically. d) In stacked columns corpses growing no fungi are in white, unknown fungi in black, A. fumigatus in blue, and A. nomius in yellow

Table 1. GLM results showing the effect of gaster-liquid treatment (from ants collected from nest plates with and without an ant corpse) and sugar control on the spore germination of *F. solani* and *A. nomius* (in comparison to control samples).

F.solani	**Estimate**	**SE**	**t**	**p**
Intercept	102.79	4.22	24.39	<0.001
Gaster-liquid, corpse	-66.95	3.56	-18.822	<0.001
Gaster-liquid, no corpse	-65.06	3.56	-18.29	<0.001
Control, sugar	2.00	3.65	0.55	0.589
Plate	-6.25	1.56	-3.99	<0.001
A.nomius	**Estimate**	**SE**	**t**	**p**
Intercept	96.85	5.90	16.41	<0.001
Gaster-liquid, corpse	-55.68	5.06	-11.01	<0.001
Gaster-liquid, no corpse	-55.01	5.06	-11.88	<0.001
Control, sugar	-2.01	5.06	-0.39	0.69
Plate	-3.75	2.17	-1.73	0.09

References

1. Viljakainen, L. *et al.* Social environment affects the transcriptomic response to bacteria in ant queens. *Ecol. Evol.* **8**, (2018).

2. Therneau, T. M., Grambsch, P. M. & Pankratz, V. S. Penalized survival models and frailty. *J. Comput. Graph. Stat.* (2003). doi:10.1198/1061860031365
